# Case report: Successful treatment of OXA-23 *Acinetobacter baumannii* neurosurgical infection and meningitis with sulbactam-durlobactam combination therapy

**DOI:** 10.3389/fmed.2024.1381123

**Published:** 2024-05-15

**Authors:** Jacob W. Snowdin, Nicholas J. Mercuro, Michael P. Madaio, Stephen A. Rawlings

**Affiliations:** Department of Infectious Diseases, Maine Medical Center, Portland, ME, United States

**Keywords:** *Acinetobacter baumanii*, carbapenem-resistant *Acinetobacter baumanii*, sulbactam-durlobactam, nosocomial infection, meningitis

## Abstract

Meningitis caused by *Acinetobacter* species is a rare complication of neurosurgical procedures, although it is associated with high morbidity and mortality. Carbapenem-resistant *Acinetobacter* is particularly difficult to treat, considering the limited selection and tolerability of effective antimicrobials. Sulbactam-durlobactam was approved by the FDA in 2023 for treatment of hospital-acquired and ventilator-associated pneumonia due to susceptible strains of *Acinetobacter*, including carbapenem-resistant *Acinetobacter baumannii*. Here, we present a case of carbapenem-resistant *Acinetobacter baumannii* neurosurgical infection and meningitis successfully treated with sulbactam-durlobactam combination therapy.

## Introduction

Neurosurgical infection and meningitis are rare but serious complications following cranial procedures. In nosocomial infections, resistant Gram-negative bacilli such as *Acinetobacter baumannii* are often implicated and are associated with mortality upwards of 40% (1). With an already limited antimicrobial armamentarium against carbapenem-resistant *A. baumannii* (CRAB), medical management becomes difficult considering the efficacy, tolerability, and pharmacokinetics of available antibiotics in the setting of central nervous system (CNS) infections. A multifaceted approach that includes supportive care, surgical source control, combination antimicrobial therapy, and extensive rehabilitation are needed to achieve successful treatment and recovery.

In the most recent iteration of the IDSA nosocomial meningitis and ventriculitis guidelines, the only treatment recommended for CRAB is colistin which does not readily enter the cerebrospinal fluid (CSF) (2). Efficacy data for treatment of CNS infections due to CRAB with parenteral antibiotic therapy is limited. We report a case of CRAB craniectomy site infection with concomitant meningitis successfully treated with IV sulbactam-durlobactam (SUL-DUR) combination therapy.

## Case report

**Figure 1:**
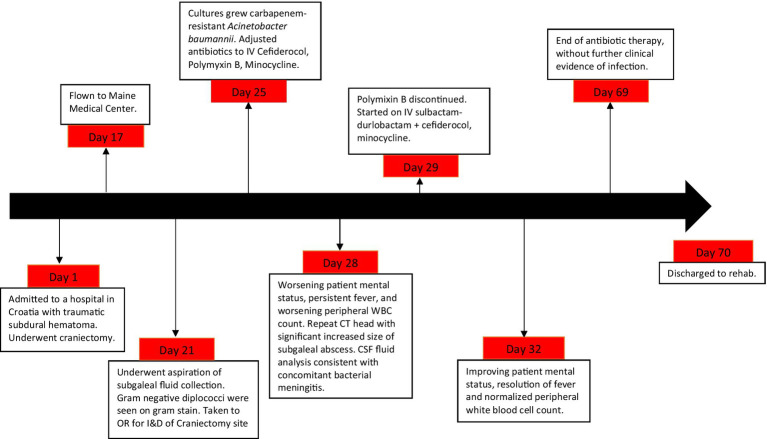
Timeline diagram of disease and treatment course.

A 75-year-old woman from the United States was brought to a hospital in Croatia, where she was found to have a left-sided subdural hematoma after suffering a traumatic fall while vacationing. She underwent emergent left hemicraniectomy and was admitted to intensive care for several days. She subsequently developed subgaleal hematoma, requiring surgical evacuation and drain placement. After 17 days, she was transferred to Maine Medical Center (Portland, ME). On hospital day 4 of admission, she developed fevers, altered mental status, and swelling at her craniectomy site. She was taken for incision and drainage of her craniectomy site, with intraoperative findings of infected epidural fluid collection. The Gram-stain from the fluid collection revealed gram negative diplococci and bacterial culture grew an OXA-producing *Acinetobacter baumannii*, (detected by Verigene Nanosphere®) later confirmed to be OXA-23 by gene sequencing. She was treated with combination cefiderocol (susceptible by Kirby-Bauer disc diffusion), minocycline (MIC = 8 mg/L, BD Phoenix®), and polymyxin B (MIC = 0.5 mg/L, broth microdilution). Despite efforts at surgical source control and combination antibiotic therapy, her mental status continued to decline with increasing peripheral leukocytosis after several days. Her exam was consistent with worsening delirium – She was non-verbal, agitated, and not interactive. A repeat CT scan of the head demonstrated an enlarging fluid collection at her craniectomy site. A bedside aspiration of CSF was cloudy with 1,724 WBC/mm3 (95% polymorphonuclear cells), glucose 29 mg/dL, and a protein concentration greater than assay, consistent with concomitant bacterial meningitis, although CSF bacterial culture did not yield growth. Polymyxin B was discontinued due to concern for nephrotoxicity, volume overload, and unachievable pharmacodynamics in the CNS space. Expanded access intravenous (IV) sulbactam-durlobactam (SUL-DUR) 2 grams every six hours was started (MIC = 4 mg/L, broth microdilution), and cefiderocol and minocycline were continued in combination for the epidural empyema and meningitis. Susceptibility and synergy testing (Table 1) with SUL-DUR was performed by Entasis Laboratories (Waltham, MA). Fevers, leukocytosis, and mentation gradually improved. Minocycline was stopped after 4 weeks due to elevated hepatic enzymes (ALP 515, ALT 90, AST 70), which quickly normalized after discontinuation of this drug. At the end of a 6-week course of SUL-DUR, a repeat CT scan of the head showed decreased size of the fluid collection the patient had clinical resolution of infection. All antibiotics were stopped and she was discharged to a rehab facility. She has since made a complete recovery with no further complications (Figure 1).

**Table 1:** Susceptibility of OXA-23 producing *Acinetobacter baumannii* isolate.

Antimicrobial	MIC (mg/L)	Determination method
Amikacin	>32	
Ampicillin/sulbactam	>16/8	
Cefepime	>16	
Ceftazidime	>16	
Ceftazidime-avibactam	>128	Etest
Ceftolozane-tazobactam	>8/4	Phoenix Emerge
Cefiderocol	S 1	Kirby bauer disc diffusion Broth microdilution
Ciprofloxacin	>2	Phoenix Emerge
Colistin	0.5	Broth microdilution
Gentamicin	>8	Phoenix Emerge
Imipenem-relebactam	>32	Etest
Levofloxacin	>4	
Meropenem	>32	Etest
Minocycline	8	
Tigecycline	4	
Sulfamethoxazole-trimethoprim	>2	
Sulbactam-durlobactam (fixed 4 mg/L) With meropenem (1:1) With cefiderocol (1:1) With tigecycline (1:1) With cefiderocol and tigecycline (1:1:1)	4 4 1 2 0.5	Broth microdilution

## Discussion

Infections due to CRAB remain difficult to treat, with few therapeutic options. The IDSA guidance for treatment of antibiotic resistant Gram-negative infections suggests combination therapy with at least two active agents for treatment of CRAB infections, noting lack of robust data to support this practice. Use of high-dose ampicillin-sulbactam with at least one other agent is suggested, a recommendation that preceded the approval of SUL-DUR. This practice considers the anticipated high bacterial burden of CRAB due to delays in initiation of effective antimicrobial therapy, and potential of this organism to develop resistance to a single agent that had previously demonstrated *in vitro* activity (3). In a randomized clinical trial evaluating treatment response to antimicrobials, 39 patients diagnosed with VAP due to CRAB were randomly assigned to receive either colistin monotherapy or colistin plus high-dose ampicillin-sulbactam. A positive treatment response at day 5 was observed in 70% of patients treated with colistin and ampicillin-sulbactam combination therapy, compared to 15% treatment response in the colistin monotherapy group. Additionally, in patients experiencing treatment failure on colistin monotherapy, 46% of patients demonstrated subsequent treatment response when ampicillin-sulbactam was added (4). Conversely, the largest combination therapy study on CRAB (primarily) to date did not find any clinical benefit with the addition of meropenem at standard doses to colistin, compared to colistin monotherapy (5).

The treatment of meningitis or ventriculitis due to CRAB represents a unique challenge, considering antimicrobial selection is further limited to agents that penetrate the CNS and achieve therapeutic CSF concentration (Table 2). Historically, the most reliable strategy for achieving therapeutic antibiotic concentrations in CSF for CRAB infections was direct instillation to the site of infection, using intraventricular or intrathecal administration with polymyxins or aminoglycosides. Agents that are typically reliable from a pharmacokinetic basis for treating meningitis, such as fluoroquinolones and trimethoprim-sulfamethoxazole, demonstrate limited susceptibility to CRAB, at 1.3 and 17% (respectively) in the JMI public database (6). While minocycline retains activity, the MIC distribution (MIC50/90 of 4–8 mg/L) in CRAB would make reaching target attainment in most cases (particularly in CSF) unachievable (6).

**Table 2:** Peak CSF concentrations of antibiotics.

Agent/dosage	Highest CSF concentration detected (from reference)	CRAB MIC50 (mg/L)^6^
Sulbactam 1,000 mg IV Q6H*^10^	12 mg/L^10^	>32 (ampicillin-sulbactam)
Imipenem 1,000 mg IV Q6H* ^17^	11 mg/L^17^	>8
Colistin 225 mg IV Q24H ^18^	0.1 mg/L^18^	≤0.5
Cefiderocol 2gm IV Q6H* ^15^	25.5 mg/L^15^	0.25
Minocycline 200 mg IV Q12H*^19^	1 mg/mL^19^	4
Tigecycline 50 mg Q12H* ^20^	0.048 mg/L^20^	2
Tobramycin 80 mg*^21^	1.35 mg/L ^21^	>8
Sulfamethoxazole-trimethoprim 5 mg/kg TMP and 25 mg/kg SMX infused over 1 h ^22^	TMP 3.2 mg/L, SMX 40 mg/L^22^	>4
Levofloxacin 500 mg q12H* ^23^	1.99 mg/L^23^	>4

Carbapenem resistance is associated with higher mortality in patients with meningitis due to Acinetobacter (7, 8). In patients with meningitis or ventriculitis due to CRAB, use of colistimethate sodium or polymyxin B administered by IV and intrathecal route is recommended, as entry into the CSF is highly limited (2). Colistimethate-containing regimens administered concomitantly via IV and intrathecal/intraventricular routes have demonstrated improved cure and survival in patients with meningitis or ventriculitis due to CRAB (8). However, elevated risks for nephrotoxicity and chemical meningitis associated with their use highlights a desire for safer alternatives (9). Evidence for efficacy of ampicillin-sulbactam for meningitis due to CRAB is limited with mixed results in the literature, and sulbactam CNS penetration is dependent upon the degree of meningeal inflammation (10). Jimenez-Mejias et al. reported clinical cure in 6 of 8 patients with meningitis due to multidrug-resistant Acinetobacter using ampicillin-sulbactam, of which 7 isolates were resistant to imipenem (11). Sun et al. reported successful treatment using ampicillin-sulbactam in 11 of 12 cases of meningitis due to MDR *A. baumannii.* Two isolates were intermediately susceptible to ampicillin-sulbactam, treated with concomitant intrathecal amikacin (12).

SUL-DUR is a co-formulated non-beta-lactam, beta-lactamase-inhibitor (BL-BLI) combination designed to primarily inhibit OXA-carbapenemases (DUR) in *A. baumannii*, and bind to PBP1 and PBP3 (SUL). Resistance to SUL in Acinetobacter sp. is generally caused by inactivation via beta-lactamases such as OXA-23, OXA-24/40, TEM-1, and KPC, or even mutations in PBP3 (1). Durlobactam (formerly ETX2514) is the diazabicyclooctanone beta-lactamase inhibitor component which can also bind to PBP2; in combination with sulbactam, susceptibility improved from 37% (SUL) to 98% (SUL-DUR) in a global survey of 4,038 *A. baumannii* isolates. In a randomized trial (ATTACK) including patients with pneumonia caused by *A. baumannii*, SUL-DUR in combination with imipenem was non-inferior to colistin in respect to all-cause mortality (19% vs. 32%) and also had better clinical response, cure, and safety (in respect to acute kidney injury) (13). Imipenem was used as a background therapy in the ATTACK trial; however, a carbapenem did not enhance the potency of SUL-DUR in our patient’s isolate while the MIC decreased 3-fold in combination with cefiderocol and tigecycline. Conversely, in a randomized trial comparing cefiderocol to best available therapy, treatment failure and mortality was greater in the cefiderocol arm in a subset of patients with *A. baumannii* infections (14). Additionally, the performance of cefiderocol susceptibility testing represents an additional challenge to its use in clinical practice for CRAB infections. Disc diffusion appears to be a valid method of determining susceptibility (15), while EUCAST has issued warning against use of certain broth microdilution platforms for determining cefiderocol susceptibility (16). Cefiderocol and sulbactam readily penetrate the CSF in patients with bacterial meningitis and significant inflammation (10, 17, 18). At 2 grams every 6 h administered IV (infused over 3 h), cefiderocol peak concentrations in the CSF have been shown to reach 24–25.5 mg/L (17), while sulbactam CSF concentrations can reach 12 mg/L at 1 gram every 6 h administered IV (10). However, the pharmacokinetics of durlobactam entry into CNS tissues are relatively unknown.

## Conclusion

In summary, there is limited efficacy data and treatment options for CNS infections due to CRAB. Despite attempts at surgical source control and aggressive antimicrobial combination therapy, our patient exhibited progressive clinical decline with evidence of worsening infection and severe meningitis. After adjustment to an antimicrobial regimen containing SUL-DUR, the patient exhibited a clear response to therapy, and in due course, clinical cure of her infection. Further studies describing the pharmacokinetics of SUL-DUR entry into the CNS are needed.

## Data availability statement

The original contributions presented in the study are included in the article/supplementary material, further inquiries can be directed to the corresponding author.

## Ethics statement

The studies involving humans were approved by Maine Medical Center Institutional Review Board. The studies were conducted in accordance with the local legislation and institutional requirements. The participants provided their written informed consent to participate in this study. Written informed consent was obtained from the patient for the publication of this case report.

## Author contributions

JS: Conceptualization, Visualization, Writing – original draft, Writing – review & editing. NM: Conceptualization, Data curation, Writing – original draft, Writing – review & editing. MM: Conceptualization, Investigation, Writing – original draft, Writing – review & editing. SR: Conceptualization, Supervision, Writing – original draft, Writing – review & editing.
